# Bilingual Object Naming: A Connectionist Model

**DOI:** 10.3389/fpsyg.2016.00644

**Published:** 2016-05-09

**Authors:** Shin-Yi Fang, Benjamin D. Zinszer, Barbara C. Malt, Ping Li

**Affiliations:** ^1^Department of Psychology and Center for Brain, Behavior, and Cognition, The Pennsylvania State UniversityUniversity Park, PA, USA; ^2^Department of Brain and Cognitive Sciences, University of RochesterRochester, NY, USA; ^3^Department of Psychology, Lehigh UniversityBethlehem, PA, USA

**Keywords:** computational modeling, self-organizing map, bilingual lexicon, object naming, semantic convergence

## Abstract

Patterns of object naming often differ between languages, but bilingual speakers develop convergent naming patterns in their two languages that are distinct from those of monolingual speakers of each language. This convergence appears to reflect interactions between lexical representations for the two languages. In this study, we developed a self-organizing connectionist model to simulate semantic convergence in the bilingual lexicon and investigate the mechanisms underlying this semantic convergence. We examined the similarity of patterns in the simulated data to empirical data from past research, and we identified how semantic convergence was manifested in the simulated bilingual lexical knowledge. Furthermore, we created impaired models in which components of the network were removed so as to examine the importance of the relevant components on bilingual object naming. Our results demonstrate that connections between two languages’ lexicons can be established through the simultaneous activations of related words in the two languages. These connections between languages allow the outputs of their lexicons to become more similar, that is, to converge. Our model provides a basis for future computational studies of how various input variables may affect bilingual naming patterns.

## Introduction

### Lexical Categorization in Different Languages

In today’s globally connected world, more people are learning a second language (henceforth L2) than at any other time before. However, acquiring native-like word knowledge of an L2 is not simply to match (or translate) the known lexical items of the first language (L1) to those of the L2. This is because languages differ in the way that words or names are mapped onto objects (see Malt and Majid, 2013 for review). Although technology has made it easy to translate words from one language to another^1^, these translations often do not accurately represent the different word-object mapping relations in different languages. For example, *table* can be translated to [scale=.60]./fig/img001.eps in Chinese, *mesa* in Spanish, *Tisch* in German, *stół* in Polish, and *tavolo* in Italian. However, even for such simple cases, there may not be one-to-one correspondences between L1 and L2: In Polish, there is no single term that corresponds directly to *table* in English (Wierzbicka, 1992; Polish *stolik* refers to coffee table and *stół* to dining room table); in Spanish, fish is referred to as either *pez* (live fish) or *pescado* (fish as food). Countless such examples exist across languages.

Thus, learning the meanings of L2 words as used by monolingual speakers of the language implies more than simple translations from L1 to L2; native-like L2 learning involves understanding the mapping relationships between objects and their names in each language. Bilinguals, however, do not necessarily acquire two sets of word meanings equivalent to those of monolingual speakers of the L1 and the L2, as we discuss below. The goal of the current research is to provide and test a computational model that captures bilingual word representations and provides a foundation for future investigations into the variables that affect the output (name choices) generated by such representations.

### Lexical Categorization in Bilinguals

The complex mapping relationships between objects and names in different languages pose a challenge to speakers of two languages. Several empirical studies have investigated the object naming patterns of bilinguals and how bilinguals deal with these inconsistent mapping relations. For example, Malt and Sloman (2003) found that sequential bilinguals who learned English as L2, even after many years of immersion in an English-language environment, still showed patterns different from native English speakers in naming household containers and dishware (e.g., plates and cups). Recent investigations have focused on further characterizing the nature of bilingual lexical representations and the factors that drive the particular naming patterns that emerge. Zinszer et al. (2014) examined variables that could help determine the conditions under which second language learners show better or worse mastery of the second language naming patterns. One important variable was name agreement: the proportion of native speakers who agree on the dominant name for a particular object. In picture naming studies, name agreement has a significant impact on naming latency (Kremin et al., 2000), event-related potentials (Cheng et al., 2010) and functional brain response (Kan and Thompson-Schill, 2004), implying that objects with high name agreement have stronger object-name associations and more robust representations. Zinszer et al. (2014) found that the name agreement levels in both L1 and L2 play an important role in L2 naming patterns. In addition, learner characteristics such as age of immersion mattered, suggesting complex interactions between languages in the acquisition of L2 vocabulary, particularly with regard to object naming patterns in L1 versus L2. In another recent study, Malt et al. (2015) found that Mandarin–English bilinguals who arrived in an English-speaking environment after age 15 showed different English word use patterns from monolingual English speakers. With increased language experience and proficiency, the bilinguals’ naming patterns moved closer to those of English monolinguals. However, higher English usage was associated with more discrepancy in L1 word use from monolingual Mandarin speakers. These findings suggest that the lexical network remains plastic into adulthood, producing dynamic patterns of cross-language interactions even with late L2 learning.

The current study builds on the empirical findings and approaches of two related studies: Ameel et al. (2005, 2009). Ameel et al. (2005) found that adult simultaneous bilingual speakers of Dutch and French developed patterns of naming that differ from the patterns of monolingual speakers of either language, suggesting that the bilinguals’ lexical representations reflect the convergence of two languages. They compared the word use patterns of adult Dutch–French simultaneous bilinguals to those of monolingual Dutch and French speakers in Belgium. The participants named pictures of common household objects, as in Malt et al. (1999). In one analysis, Ameel et al. (2005) examined the most frequent names produced by the participants for each object. In a second analysis, the full name distributions for each object were used, in order to take into account the entire range of responses to each object and the frequency of each response (see Model Assessment and Comparison for further details of how dominant names and distribution of naming patterns were calculated). Both analyses showed that naming patterns between monolingual Dutch and French speakers were different. For the simultaneous bilinguals, the naming patterns in Dutch and French were more similar than the corresponding Dutch and French monolingual naming patterns were. That is, they converged toward each other.

Using the same data, Ameel et al. (2009) compared the category centers *between* language groups and category boundaries *within* language groups to examine if the bilinguals’ semantic convergence was manifested in the centers and/or at the boundaries of lexical categories. Category centers (prototypes) were estimated from typicality ratings and from geometrical representations (multi-dimensional scaling solutions; MDS) based on similarity judgments of the objects. Ameel et al. (2009) found that the centers of categories for translational equivalents were closer in the naming patterns of the bilinguals than those of the monolinguals. They also examined whether this higher similarity for bilinguals was due to the convergence of entire categories or the convergence of boundary exemplars specifically. The geometrical coordinates of each object from the MDS analysis provided distance information between the category centers. Their results suggested that both prototypes and category boundaries contributed to the convergence. Ameel et al. (2009) further evaluated how the category boundaries within a language manifested convergence. They used a test of linear separability to determine the number of the dimensions (based on the MDS analyses) needed to separate two categories as the index of complexity at category boundaries. They found that the number of dimensions needed to separate categories was significantly fewer for bilinguals than for monolinguals, suggesting that bilinguals have less complex category structures and that exemplars at the boundaries of the categories are more likely to be named based on their similarity to the prototypes. To further test this possibility, Ameel et al. (2009) also compared the proportion of outliers (i.e., number of outliers divided by number of objects for a category) between bilinguals and monolinguals. Outlier objects were identified as those objects closer to another category center than to their own category center. Consistent with the preceding result, the proportion of outliers was smaller for bilinguals than for monolinguals.

### Computational Modeling of Bilingual Lexicon

The study of the dynamic interactions in the representation of two lexical systems lends itself naturally to connectionist representation and modeling. We can think of a lexical network in the bilingual mind in terms of conceptual and word-form representations. The conceptual representations are composed of features that describe each object (e.g., several cups with slightly different features) and the relationships between objects are based on feature associations in a connectionist network.

One way that languages might influence each other is via the associative connection weights that hold between features of the word meaning and the word form. When a new L2 word form is taught as a translation equivalent of an L1 word, the network will set initial weights to match those of the L1 word. The L2 word will be activated by the same features as the L1 word, and non-native L2 patterns of production will result. Over time, however, these weights will be modified by L2 experience and will move away from a uniform pattern driven by L1. The weights may settle into a pattern that does not exactly match that of the L2 but reflects the convergence of L1 and L2. In such an account, the meanings associated with word forms are different for the bilingual compared to those of monolingual speakers of the two languages.

Another possible form of influence can come from the associative connections of weights between two languages on the word form level (i.e., lateral connections). These connection weights are first established after the L2 word form is taught. De Groot (2014) suggested that observed bilingual convergence in phonology, grammar, and word use may not reflect convergence in underlying representations in the two languages. Instead, it may reflect connections between two sets of perfectly monolingual-like representations such that cross-activation of representations of the two languages results in one language having an influence on the behavioral output in the other. Thus, when a new L2 word form is taught as a translation equivalent of an L1 word, the word forms are linked, and activation of a word in one language will create activation to the associated word in the other language.

The computational model we presented in this study will specifically address how word-form level lexical activations, along with the lateral connections between the bilingual word forms, might impact lexical representations, and consequently, naming patterns in the two languages. We compare models that have such cross-language lateral connections with models that do not. Computational models implemented in this fashion allow us to capture cross-language lexical interactions because of their ability to manipulate learning conditions, including important learner and input characteristics such as age of exposure, proficiency in each language, frequency of input, and similarities between the lexical items. Such models will also make it possible to examine the learning trajectory (instead of just the mature state) so that we can identify how naming patterns change as a result of the shift in the relative dominance of first versus second language. Although models that systematically manipulate these learning conditions for bilingual naming remain to be developed, in this study we make an initial effort to begin with a computational model based on self-organizing feature maps to study cross-language lexical interaction in bilinguals.

### Self-Organizing Map Models of Language Representation

The self-organizing map (SOM) model has been an enabling approach to the study of monolingual and bilingual language representation in recent years (see Li, 2013; Li and Zhao, 2013, 2015 for reviews). SOM is a type of unsupervised learning (Kohonen, 2001) that extracts and represents similarities in the input by projecting complex, high-dimensional, vector-based patterns onto a two-dimensional space to produce a topographical structure. Because of this dimensionality-reduction ability, SOM is a powerful tool to visualize the complex relationships between input patterns in a 2D space. For each input vector, the SOM identifies a node that is most similar to the input vector as the Best Matching Unit (BMU), and adjusts the weights of the BMU so that over time, it serves as a unique representation of that input pattern in the entire input space. In addition to adjusting the weights of the BMU, the model also adjusts the weights of the BMU’s neighbors using a Gaussian kernel. As training progresses, the weight vectors of the BMU and its neighboring nodes become more similar to the input vector. Subsequently, similar input vectors will end up being represented by nodes near one another (i.e., a BMU and its neighbors) in the SOM’s topographical space.

Self-organizing map-based connectionist models have been applied successfully in past research to the study of both first and second language lexical development and processing (See Miikkulainen and Kiran, 2009; Zhao and Li, 2010, 2013; Kiran et al., 2013; Shook and Marian, 2013; see Li and Zhao, 2013, 2015 for recent reviews). For example, Zhao and Li (2013) built a three-layer SOM-based model and simulated cross-language priming asymmetry whereby cross-language lexical priming is stronger from L1 to L2 than from L2 to L1. The computational model, unlike previous qualitative models, provided a mechanistic account for priming asymmetry and for important learner variables (e.g., age of L2 acquisition) that contribute to cross-language lexical competition (priming from L1 to L2 words and vice versa). These effects arose in the model because of the computational implementation of distributed and overlapping semantic features within and across languages. In another study, Kiran et al. (2013) applied SOM to simulate bilingual language disorders and recovery in aphasic patients. Their model was the first to map a SOM-based model to individual behavioral patterns of 17 aphasic patients on a case-by-case basis, and the model was able to predict individual patients’ success in rehabilitation. Computational studies of this type allowed the researchers to compare a lesioned model not only with an injured brain, but also with an intact model before lesion (i.e., the patient’s pre-damage state, something that cannot be reversed in real life). They demonstrate the utility of SOM-based computational models in simulating variation and development, offering explicit, mechanistic accounts of bilingual language processing.

The goal of the present study is to build a SOM-based computational model that can capture patterns of bilingual lexical categorization and help identify the computational mechanisms underlying bilingual lexical semantic convergence. Through simulations and model comparison, we examined factors that are potentially important in bilinguals’ lexical categorization, including the role of orthographic and phonological overlap between languages and, most critically, lateral connections between word forms. As a first connectionist model of bilingual lexical categorization, it focuses on describing the dynamic changes in the semantic convergence of naming patterns as a result of learning and representing both languages.

## The Model

### Model Architecture

**FIGURE 1:**
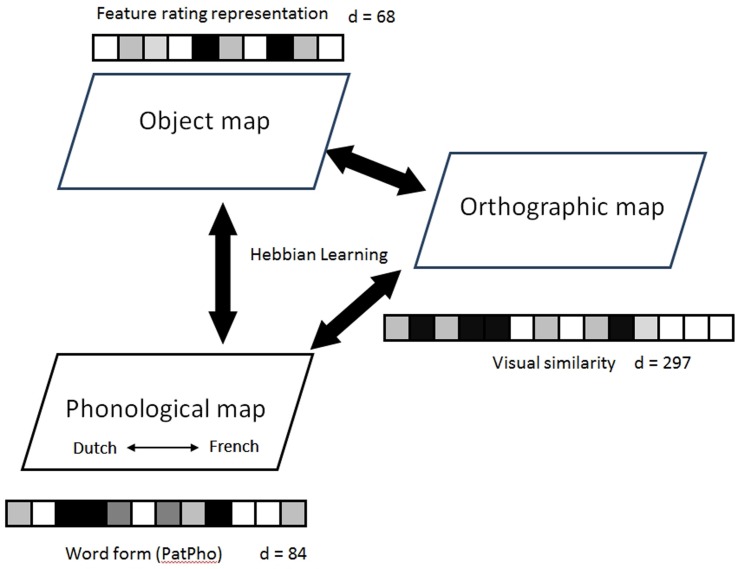
**The architecture of the standard model.** Each of the three self-organizing maps (SOM) takes the monolinguals’ naming data from Ameel et al. (2005) as its input and organizes semantic, phonological, and orthographic information, respectively. Each map has 30 × 40 nodes. The dimension of the input vector for each map is indicated by “d =”. The maps are connected via associative links updated by Hebbian learning. See text for further explanation of the model.

As discussed above, the current study uses a SOM-based connectionist model to examine bilingual lexical representations and the corresponding naming patterns. **Figure 1** presents a diagrammatic sketch of our model. The model is a multi-layer SOM neural network model, which includes three basic SOMs (i.e., semantic^2^, phonological, or orthographic). The SOMs are connected via associative links updated by bi-directional Hebbian learning (Hebb, 1949). All three SOMs were implemented on a two-dimensional grid (Kohonen, 1982). Each node on the grid consists of a high-dimensional weight vector. The number of dimensions is based on empirical data described below.

As in Zhao and Li (2010, 2013), our model uses the same SOM for representing both languages at each level, rather than separate SOMs for each language (as in Miikkulainen and Kiran, 2009). That is, we trained lexical items from both languages within the same SOM and did not manually label these items as belonging to one or the other language as would be required if separate SOMs were used. This approach is justified especially in light of current evidence from bilingual brain studies, which indicate that bilingual learners rely on convergent neural resources to handle both languages (Abutalebi and Green, 2007; Li et al., 2014). Previous work has suggested that training both languages in the same SOM will result in localized clusters for each language and intersecting boundaries that contain words of high representational similarity, even without explicit labeling of the language membership of lexical items (Li and Farkas, 2002; French and Jacquet, 2004; Shook and Marian, 2013; Zhao and Li, 2013). However, previous work has not explicitly modeled a production (object naming) task on a word-by-word basis (given the large lexicon size in those studies). Moreover, in previous modeling of language comprehension, labels of language membership (whether a word is from Language A or Language B) as well as the labels for individual words, were often assigned to the L1 and L2 lexicons (e.g., Zhao and Li, 2010). In the current study, we discriminate between representations in each language only in the context of the production task, and no labels were given to the representations a priori during the learning process.

#### The Semantic SOM

The semantic SOM was the core of the model, implemented to represent the physical and functional properties of the objects to be named. This SOM was trained using input vectors with weighted object features generated from human responses. The training approach used was similar to the feature-based method as in Ritter and Kohonen (1989), which generates the meaning of words. In the feature-based method, each value in the vector was between 0 and 1, indicating the degree of absence (0) or presence (1) of a particular feature for the target word. In our simulation, in addition to objective object features (e.g., ‘it is made of glass’), we also used subjective object features (e.g., ‘it is deep or you can put something in it’). Both the objective and subjective features were provided by Ameel (unpublished data; personal communication, 2015) and feature vectors were generated as input representations to the model.

The feature vector representations were generated using the three-step procedure of Ameel and Storms (2006), as reported in Ameel et al. (2008): feature generation, feature matrix formation, and matrix filling. During feature generation, 50 Dutch speakers^3^ (5-, 8-, 10-, 12-, 14-year-olds and adults, 10 of each group) were asked to list properties for the six names that covered most of the stimuli. Participants were encouraged to generate as many different features as they could for each name. To stimulate generation, they were prompted with questions including “What are – in general – the features of a typical X?”, “What makes something a typical [or borderline] X?” and “Why are some things better exemplars of a category than others?” During feature matrix formation, the generated responses were cleaned and organized as in McRae et al. (1997). The procedure included combining synonymous features to produce unique features, dropping quantifiers (e.g., ‘most of them are transparent’ → “are transparent”), extracting distinct features from adjective–noun combinations (e.g., ‘are made of white paper’ → “are made of paper” and “are made of white paper”) and conjunctive features (e.g., ‘is long and wide’ → “is long” and “is wide”). A master list of properties/features was compiled consisting of all features listed by at least two participants for a given name in a given age group. Finally, during the matrix filling step, twelve additional adults were asked to assign a 1 (yes) or a 0 (no) to indicate whether or not a feature (from the above two steps) applied to each object. (See Ameel et al., 2008, p. 274 for further details of the process.) The semantic vector was calculated as the number of all yes responses from the 12 adults for each object. A total of 68 features were generated and incorporated, so we created a 68-dimension feature vector for each of the 73 objects. Note that these features formed natural clusters that reflect the grouping criteria for the containers, for example: “you can unfold it” for action cluster, “it has a lid” for external component cluster, and “it is made of plastic” for material cluster, etc.

#### The Phonological SOM

The phonological SOM was implemented to represent the phonological properties of the object names. Word form representations were trained in the SOM using vectors generated by PatPho, a generic phonological pattern generator for neural networks (Li and MacWhinney, 2002; Zhao and Li, 2009). The phonological forms of words were composed of sequences of phonemes, obtained from dictionaries of the two target languages (Osselton and Hempelman, 2003, for Dutch; Correard and Grundy, 2001, for French). Each phoneme was coded in three dimensions: vowel vs. consonant, place of articulation, and manner of articulation, and the coding followed the International Phonetic Alphabet (IPA). Each word in the model is left-justified in a five-syllable template following the structure: CCVV/CCCVV/CCCVV/CCCVV/CCCVV/CCC, where Cs represent consonants and Vs represent vowels (the last three CCC representing the ending consonant clusters; see Li and MacWhinney, 2002). In this fashion, each word’s phonological form is made of an 81-dimension vector (three phoneme dimensions × 27 phoneme slots).

#### The Orthographic SOM

The orthographic SOM was implemented to simulate the potential role of orthography in object naming. We implemented two models, one with orthographic representations and one without, in order to identify the role that orthography might play in bilingual lexical categorization for Dutch and French.

Orthographic measures have not been traditionally incorporated in bilingual lexical categorization research. Nonetheless, several studies have suggested that orthographic information is activated when presenting pictures and it plays an important role in picture naming (Lupker, 1982; Bi et al., 2009; Zhang and Weekes, 2009; Zhang et al., 2009; Rastle et al., 2011). For example, when naming a picture with a word superimposed on it, the orthographically similar picture–word pairs were found to have faster naming speed in comparison to the unrelated pairs (Lupker, 1982). This orthographic facilitation effect has also been shown in Chinese in which phonological and orthographical information can be manipulated independently and possibly accessed without the mediated of phonology in some cases (Bi et al., 2009; Zhang and Weekes, 2009; Zhang et al., 2009). Rastle et al. (2011) designed a training study to examine the effect of orthography on speech production using well controlled spelling-sound regularity. They trained participants with a task of association of novel pictures with spoken words. In addition to the association task, participants also had been introduced to the spelling of the words. Rastle et al. (2011) found that after training, participants’ picture naming speed was influenced by the spelling regularity, which suggested that orthographic representations may become activated automatically during picture naming and there are bi-directional flows of activation between orthographic and phonological representations. Finally, studies have shown that cognates play a special role in bilingual word recognition, production, and learning. In picture naming, pictures that are described by cognates were named faster than those for non-cognates (Costa et al., 2000) and orthographic overlap between items is necessary to obtain robust cognate priming effect (Bowers et al., 2000). The role of orthographic similarity in bilingual lexical convergence may be particularly important for languages that have cognates, as Dutch and French do.

The orthographic SOM was trained using vectors that describe the pixel patterns for images of each alphabetic character in a word. This method is similar to that used by Miikkulainen (1997) and Shook and Marian (2013). Each Dutch and French alphabet (the 26 alphabetic characters and è, é, and î) was typed in 12 point, Arial font in black on a while background measuring 90 × 90 pixels. Each character image was divided into nine cells (3-by-3, each cell has 900 pixels [30 × 30]). The proportion of black pixels in each cell (i.e., number of black pixels/900) was calculated and used to create a 9-dimension vector for each letter within a word. The letters were then concatenated into a 33-slot template of the form CCCVVV/CCCVVV/CCCVVV/CCCVVV/CCCVVV/CCC for a word. Each orthographic entry thus consisted of a 297-diminsional vector. For example, the Dutch word, *bidon*, was coded as b– –i– –/d– –o– –/n– – – – –/– – – – – –/– – – – – –/– – –, in which dashes (“–“) are zeros in the vector representing the word, and the vectors for letter b and d are [0.23 0.23 0.08 0.26 0.12 0.22 0.01 0.18 0.01] and [0.01 0.17 0.00 0.23 0.13 0.22 0.23 0.23 0.08], respectively.

#### Lateral Connections

Lateral connections have been shown to play an important role in the neocortex, and computational models of the primary visual cortex have relied on lateral connections (see Sirosh and Miikkulainen, 1994 for a computational implementation). Zhao and Li (2013) used lateral connections successfully to simulate cross-language priming, and Shook and Marian (2013) used lateral connections to simulate competition between languages in speech comprehension. Many studies have shown that phonological representations from both languages may be activated when bilinguals read in only one language, due to parallel bilingual lexical activation (Dijkstra et al., 1999). Through lateral connections, lexical items, both within and across the two languages, can be linked to enter into cooperation or competition regardless of their physical distance on the SOM (see Zhao and Li, 2013 for discussion).

**FIGURE 2:**
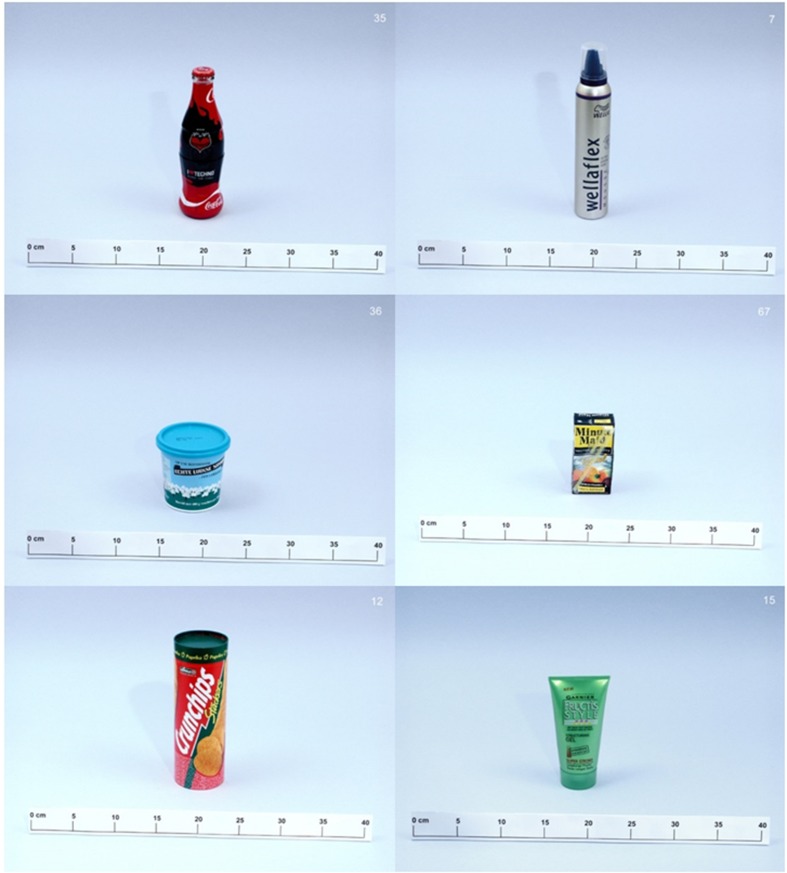
**Illustrations of some of the exemplars of the bottle set**.

In our model we assume that when an object is presented to the semantic SOM, its names in both languages will be activated on the phonological SOM through the Hebbian connections between the semantic and phonological maps. Lateral connection is implemented in the same SOM as follows, as in Zhao and Li (2013 ; see their **Figure 2** for illustration; see also Section “Model Training” for technical descriptions). First, let’s say that A and B are the two candidate words from the respective languages. A given object/stimulus produces parallel activation from both languages and the words A and B are both activated. These two words now become connected together, and their connection strength will increase according to the Hebbian rule (the more often they are co-actived, the stronger their lateral connections will become). The lateral connections between the activated names in each language are then strengthened according to the Hebbian learning rule. As a result, object naming in the model in either one of the two languages can be influenced by another language through these lateral connections. Thus, a key addition to the basic SOM architecture in the current work (e.g., as compared with the original DevLex-II model of Zhao and Li, 2010) is the use of lateral connections between languages to simulate cross-language links.

### Input Stimuli

In this study, the model received input based on the monolingual naming data from Ameel et al. (2005). We trained the model on 73 containers for household products used by Ameel et al. (2005) (mostly called *bottle*, *jar*, or *container* in American English). Objects were photographed in color against a neutral background with a ruler included in front of each object to provide additional size information. **Figure 2** provides some typical exemplars from Dutch monolinguals’ naming data (Ameel et al., 2005).

In Ameel et al. (2005), participants were asked to name each object giving whatever name they thought was best, and they were told it could be one word or more than one. Across participants, each object received 1 to 9 different names. For each object, name agreement was calculated as the percentage of participants naming the object with a given name. We call the name produced most often for an object its “dominant” name. For example, if an object were named *pot* by most French-speaking monolinguals, *pot* was taken as this object’s dominant name for this group. Eighty-one different (dominant plus less frequent) names were produced, 42 in Dutch and 39 in French.

### Model Training

In the simulations reported in this paper, we constructed each SOM with 30 × 40 nodes. This size of the SOM was selected based on considerations of both the model’s ability to represent the full scale of the input and the modeler’s ability to effectively analyze the simulation data. During training, the learning rate of the SOM was linearly decreased from 0.2 to 0.1 during the first 100 epochs, and it then remained at 0.1 for the rest of the training. This linear decrease of learning rate is a common practice in the training of SOM models (see Miikkulainen, 1997). The learning rate of Hebbian learning was set at 0.2 constantly. The initial radius of the neighborhood size was set at 15 and was adjusted according to the network’s learning outcome. We used a self-adjustable neighborhood function according to Li et al. (2007) rather than a fixed linear decrease of neighborhood size as used in other SOM training (see Li et al., 2007 for details and rationale). According to this method, in every 5 epochs of training the network would calculate the quantization errors on each map responding to input patterns. The quantization errors are defined as the Euclidean distances in the input space between an input pattern and the input weight vector of its BMU (Kohonen, 2001). If the average quantization errors from current 5 epochs remained similar as in the previous 5 epochs (i.e., within the 25% range of previous averaged quantization errors), the neighborhood radius would then decrease by 1.

**FIGURE 3:**
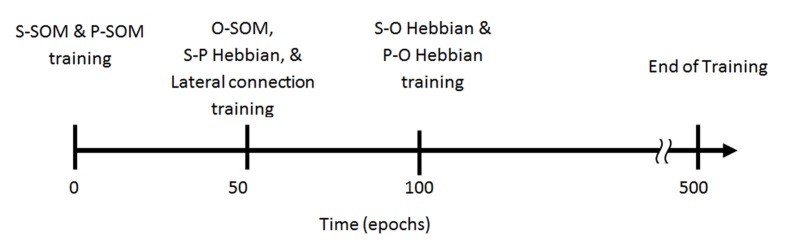
**Schematic representation of the training protocol**. S-SOM: semantic SOM; P-SOM: phonological SOM; O-SOM: orthographic SOM; S-P Hebbian: Hebbian connections between semantic SOM and phonological SOM; Lateral connections: Hebbian connections between two languages on the phonological SOM; S-O Hebbian: Hebbian connections between semantic SOM and Orthographic SOM; P-O Hebbian: Hebbian connections between phonological SOM and Orthographic SOM.

**Figure 3** depicts a timeline for the training of the model. We trained the model in three steps: (1) The semantic and phonological SOMs were trained independently (without the orthographic SOM or Hebbian connections) to simulate the learning of the properties of objects and the pronunciation of words; (2) Hebbian learning started after 50 epochs, which enabled the learning of the association between object representations and phonological forms. The orthographic SOM also started at epoch 50 to simulate the learning of written words. (3) Hebbian learning between the semantic and orthographic SOMs and between the phonological and orthographic SOMs began at epoch 100 to simulate the learning of reading comprehension and reading aloud.

The training order of the stimuli was randomly assigned at each epoch for every model. The Hebbian connections between SOMs were language specific. Although the words from both languages were represented in the same SOM, the Hebbian connections between SOMs were trained separately for each language. The names from the two languages could be the output of the same object through different Hebbian connections. The lateral connections between activated names between two languages were then strengthened by Hebbian learning rule. The Hebbian connections between the phonological and orthographic SOMs were trained with one-to-one mapping (i.e., the orthographic representation of a given word is mapped to the phonological representation of the same word). The Hebbian connections between semantic and phonological SOMs within each language (Dutch or French) were based on the monolingual naming data from Ameel et al. (2005), which were also scaled according to the name agreement scores. For example, if an object was named 81.25% as *fles* and 18.75% as *bus* in Dutch, the adjusted connection weights are calculated according to Equation (1):

(1) $${\Delta W}_{k1}={\beta \alpha }_{k}\alpha_{1}N\,$$

where Δw_kl_ is the unidirectional associative weight going from node k to node l. α_k_ and α_1_ are the activation levels of corresponding nodes. N is the name agreement (0.8125 or 0.1875 in the example), and β is a constant learning rate, which was set as 0.2. To avoid uncontrolled weight growth, a multiplicative normalization was applied to the associative weight vectors to ensure that the largest possible connection weight is no more than one (Miller and MacKay, 1994).

During the training of the Hebbian connections, the phonological SOM received input directly from the semantic SOM and indirectly from the orthographic SOM. The orthographic SOM received input from both the semantic SOM and phonological SOM and then fed back to the phonological SOM. On the orthographic SOM, only items that are orthographically similar to the target words become activated and are fed back to the phonological SOM. The level of activation of an orthographic node is the sum of activation from its neighboring activated nodes. The minimum radius of the neighborhood was set at 3 so that orthographically similar words can interact with each other in the model.

Twenty simulations of the same model with different random initial representations and weights were trained. The reported data in this paper are based on averaged results from the 20 simulations. All variations of the model were trained for 500 epochs, as illustrated in **Figure 3**. We call this the “standard” model.

### Comparison Models

In order to identify the role of orthographic information and the effects of lateral connections, we constructed two comparison models to contrast with the standard model, respectively. The comparison models are identical to the standard model in every respect, except that in each comparison model one component is removed: Comparison Model A was constructed without orthographic information and Comparison Model B was constructed without lateral connections between languages.

### Model Assessment and Comparison

We tested each simulated model at epoch 500. During testing, we presented all 73 containers to the semantic SOM and examined their activations propagating to the phonological SOM (simulating the object naming or production process). We identified the word that was named for a particular object by matching the activated nodes for that word to its phonological BMUs. The level of activation on any of given phonological BMUs was treated as the simulated name agreement. For example, if an object has activation level of 0.8 on *fles*, we call the name agreement between the object and *fles* 0.8. In order to distinguish specific language output (i.e., whether the name given was Dutch or French), we labeled the phonological BMUs with their language membership in this process.^4^

We examined each models’ naming patterns to investigate the model’s naming behavior in both languages. We also estimated the correspondence between the simulation data and empirical data in each model and compared the performances across models. Two measures were calculated: correlation of the name distributions between languages and the dominant category names.

The correlation of the name distributions indicates the extent to which the same object would elicit same or similar name distributions in each of the bilingual’s two languages (Malt et al., 1999; Ameel et al., 2005). Following Malt et al. (1999) and Ameel et al. (2005), in the first step, we constructed the name distribution for each object, consisting of a vector indicating the number of times a given name was produced for each object for a language group (monolingual Dutch, monolingual French, or bilingual Dutch–French). The size of the vector was 42, based on the total number of the different names produced for all 73 objects: for example, for one object, 11 monolingual Dutch-speaking participants called it *fles*, 10 called it *flacon* and 4 called it *pot*, and none called it by any other name. This would lead to a vector in which the dimensions for *fles*, *flacon*, and *pot* are filled with values 11, 10, 4, respectively, with the other 39 dimensions as 0s. In the second step, given the name distributions for each object for a language group, we can compute the similarity of each possible pair of objects’ naming preferences by calculating the Pearson correlations between the two objects’ distributions. There are *n*(n-1)/2 correlations, and thus 2628 correlations for the 73 objects. In a third step, we can correlate these name similarity matrices between two language groups - for example, between the monolingual groups (Dutch and French), or between each monolingual group and the bilinguals when speaking the corresponding language. In a similar fashion, we can also correlate these name similarity matrices between the simulation and the empirical data. Last, to normalize the sampling distribution of the correlations, the correlations of name distributions were converted to *Z*-value using Fisher’s r-to-z transformation (Equation 2). We compared the Fisher’s z-transformed correlations between different language groups using the *Z*-statistic as in Equation 3.

(2) $$Z^{\prime }=\frac{1}{2}\,\;\ln\left(\frac{1+r}{1-r}\right)\,$$

(3) $$Z=\frac{Z_{1}-Z_{2}}{\sqrt{\frac{1}{n_{1}-3}+\frac{1}{n_{2}-3}}}\,$$

To calculate our second measure, we looked at how well the models predicted the objects’ dominant names. As previously described, the dominant category name for each object was defined as the most frequently produced name for each object. However, some objects may elicit less consistent naming responses, especially near category boundaries (McCloskey and Glucksberg, 1978). When agreement is low, one participant’s response might determine the object’s dominant name. To avoid having the dominant name being determined by sampling noise, we used the following criterion: if the second highest name agreement score (e.g., 0.45) is more than 80% of the highest name agreement score (e.g., 0.55), both names were treated as dominant category names. The same criterion was also applied to the activation level. For each simulation, we obtained the dominant name for every input object based on the empirical naming pattern (see above). We then calculated the percentages of correct naming in each simulation to estimate the performance of the model and its match to empirical data.

While the output of the models (i.e., correlation of the name distributions and dominant names) provides data for simulating empirical findings in object naming, the model also allows us to explore the factors that influence the naming output. In our object naming simulation, the BMU with the highest activation on the phonological SOM was defined as the output naming pattern. The activation on the phonological SOM is the sum of the activations from three sources: the semantic, the phonological (i.e., lateral connection), and the orthographic SOMs. In our bilingual naming simulations, the requested output language was called the target language and the other language was called the non-target language. The activation from semantic SOM is related to the strength of name agreement for the target language which is scaled by the connection weights (Equation 1). The activation from phonological SOM is related to the connection strength between names in two languages and is modulated by the strength of name agreement of non-target language. The connection strength was established through Hebbian learning rule. If the names in each language were activated together, they resulted in stronger connections between names. Finally, the activation from orthographic SOM is constrained by the orthographic similarity between languages and modulated by the strength of name agreement. We compared the relative contributions from each source to the phonological activation and explored the factors that influence lexical naming patterns.

### Modeling Semantic Convergence

To further understand the bilinguals’ semantic convergence in their output patterns for the two languages, we followed Ameel et al. (2009) in examining the convergence effect on category centers and boundaries. The category centers for language groups (monolingual Dutch or French and bilingual Dutch–French groups) were estimated by typicality ratings and geometrical representations. The complexity of category boundaries was examined via the number of dimensions required to separate categories and the proportion of outliers for a language group (see Lexical Categorization in Bilinguals for description).

#### Category Centers: Typicality Ratings

Typicality is a measure of a particular object’s fit to its lexical category. In our simulation, we compared the magnitude of activation for each object elicited by a category name as a measure of that object’s typicality in the category. By this analogy, a higher activation level indicates greater typicality of the object for a category. If bilinguals’ category structures as reflected in the output are independent of each other in each language on the semantic map, the correlation of activation levels between each of their languages should be similar to the correlations of activation levels between each of the monolingual’s languages. On the other hand, if this correlation is higher for bilinguals, it would indicate that the categories have become more similar relative to those in the monolinguals.

To compare levels of semantic activation for different simulated language groups, analogous to Ameel et al. (2009), we used pairs of frequently generated category names that are rough translational equivalents between Dutch and French. The Dutch–French pairs selected were *fles*–*bouteille* and *pot*–*pot*. During testing, each category name (*fles*, *bouteille*, *pot*, and *pot*) was presented one at a time to the phonological SOM as input, and the level of activation propagated to the semantic map was examined. The correlations between language groups were converted to *Z*-value (Equation 2) and subjected to the *Z*-statistic (Equation 3) to test if monolinguals and bilinguals showed same level of correlation of typicality rating.

#### Category Centers: Geometrical Representation

To examine the category centers through geometrical representations, we examined the location of categories in the semantic SOM, which, like MDS, also organizes high-dimensional representations on a two-dimensional plane so that similar objects are located closer to one another on the SOM map (see earlier discussion of SOM). This feature of SOM lends itself naturally as a tool for examining the spatial relationships of category centers of different language groups. The same two pairs of roughly corresponding category names were selected and used in our simulations (*fles*–*bouteille* and *pot*–*pot*; first name in Dutch; second name in French), as in Section “Category Centers: Typicality Ratings”. The position of each of 73 objects on the 2-dimension semantic SOM was obtained by calculating the BMU of each object’s semantic representation vector from the semantic SOM. The location of category centers were computed across all the objects and weighted by the resulting magnitude of activation in the semantic SOM when presenting each of four category names to the phonological SOM. The Euclidean distances between corresponding categories were calculated to estimate if the corresponding category centers were closer for bilinguals than for monolinguals.

Analogous to Ameel et al. (2009), two types of category centers were computed: boundary-dependent and boundary-independent. The boundary-dependent center used the mean coordinates across the objects, and the boundary-independent one used the median coordinates. Thus the boundary-dependent center, compared to the boundary-independent center, would be more influenced by objects at the boundaries of the category. Both mean and median coordinates of the category were computed by weighting each object by its name activation level so that objects having high activation for a particular name would have a stronger influence than ones with weak activation for that name. By comparing the distances between category centers, we examined whether between-language categories were closer (more similar) in bilinguals than in monolinguals. This measure can also test if the findings in Section “Category Centers: Typicality Ratings” can be replicated using different approach (i.e., geometrical representation). Furthermore, by comparing between boundary-dependent and boundary-independent effects, we could examine whether boundary exemplars play an important role in the convergence.

Like Ameel et al. (2009) the relative distance among bilingual prototypes and monolingual prototypes was also calculated to investigate if bilingual prototypes would be located in between monolingual prototypes. For each pair of category names, four distances were calculated: (a) the distance between monolingual prototypes, (b) the distance between the monolingual Dutch prototype and bilingual Dutch prototype, (c) the distance between the monolingual French prototype and bilingual French prototype, and (d) the distance between the bilingual prototypes. If the bilingual prototypes are situated right in between the monolingual prototypes, the sum of distance (b), (c), and (d) will equal to the distance (a) and the ratio of the indirect distance (b+c+d) to the direct distance (a) will be equal to 1. Across 20 simulations, an independent *t*-test was conducted to examine if the ratio is significantly different from 1.

#### Category Boundaries: Complexity Level

In order to compare the complexity level of category structures between monolinguals and bilinguals, the number of dimensions required to separate two categories was used as an index of complexity level. We used a stepwise discriminant function analysis (DFA) by *SPSS* (*version 22.0*) to find the optimal number of features that could separate two categories. The DFA generates a discriminant function to predict group membership (i.e., which category that object belongs to) and produces canonical correlations to indicate the associations between the discriminant function and the predictors. The Wilks Lambda test was used to test if the discriminant function explained the group membership better than chance. The stepwise DFA selected a set of variables which best classify the group membership by adding or removing variables (here the variables are the features) until the canonical correlation was stable. We compared the number of dimensions (number of selected features in the DFA) between language groups to estimate the relative complexity of category structure.

To examine category structures within a language, Ameel et al. (2009) selected pairs of frequently generated category names in each language. In Dutch, the pairs *fles*–*bus*, *fles*–*pot*, and *bus*–*pot* were selected and in French, the pairs *bouteille*–*flacon*, *bouteille*–*pot*, and *bus*–*pot* were selected. Note that these category pairs are different from the pairs selected in Sections “Category Centers: Typicality Ratings” and “Category Centers: Geometrical Representation”. There the category pairs were selected to be rough translational equivalents between languages. Here, the category pairs were selected to examine the feature complexity that may separate categories within a language. To identify the category membership of each object, each object was presented one at a time to the semantic SOM as input, and we examined the level of activation propagated to the phonological map. The object input then generates different levels of activation for category names represented in the phonological map. For an object to be classified as having a particular category name, the category name must have the highest level of activation in response to a particular object input. For each category pair (e.g., *fles*–*bus*), if the object’s dominant name was neither of the two names in question, these objects would not be included. Sixty-eight semantic features of each object were used as independent variables. We then used a stepwise DFA to develop a discriminant function to classify each object. The stepwise procedure finds an optimal set of variables (i.e., features) for the discriminant function. The number of variables in each discriminant function was the number of the dimensions required to separate two categories and was used as the index of complexity.

#### Category Boundary: Proportion of Outliers

In order to examine whether bilinguals simplified categories by dropping language-specific naming idiosyncrasies, the proportion of outliers was calculated for each category name in each language group. The six most frequently generated category names were selected, 3 from Dutch (*fles, bus*, and *pot*) and 3 from French (*bouteille, flacon*, and *pot*) as described in Section “Category Boundaries: Complexity Level” and used in our simulation.

## Results

### Comparison between Models and Empirical Data for Bilingual Convergence

We first tested whether our model reproduced the basic bilingual naming convergence found in behavioral data by Ameel et al. (2005). If our simulated model replicated Ameel et al.’s finding, the correlations (z-transformed scores) of the name distribution matrices of the two languages in the bilingual model should be significantly higher than the correlations between those in the monolingual model.

**FIGURE 4:**
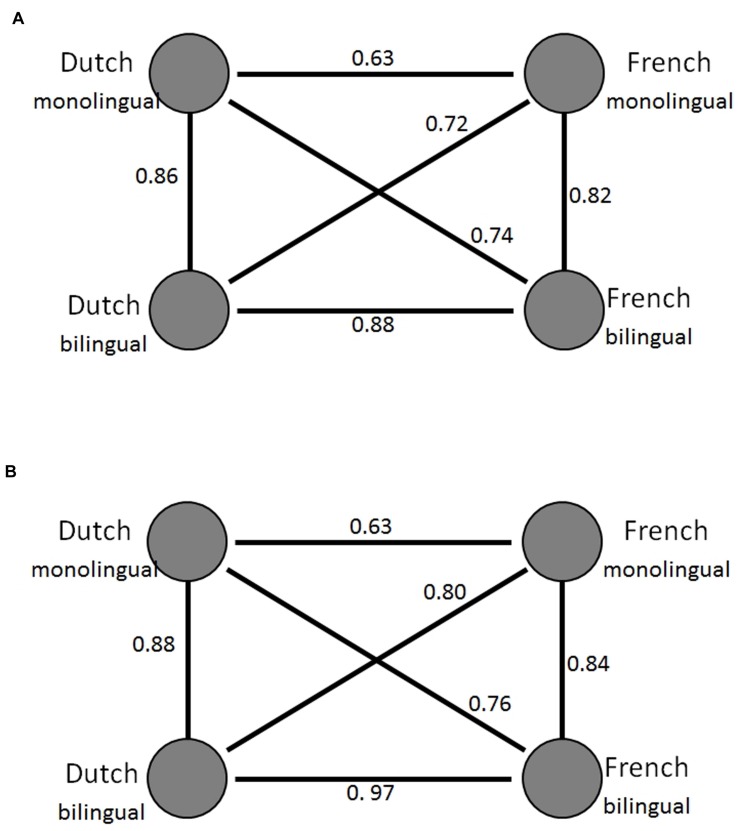
**Patterns of correlation between the name distributions of the language groups.** Dutch_monolingual_ denotes the naming patterns of the Dutch-speaking monolinguals, French_monolingual_ the naming patterns of the French-speaking monolinguals, Dutchbi_bilngual_ and French_bilingual_ the naming patterns of the bilinguals in Dutch and French, respectively. **(A)** The correlations reported in Figure 5D in Ameel et al. (2005). **(B)** The correlations from our standard model. The circles represent the naming patterns. The lines between the circles express the relations between the naming patterns. The numbers next to the lines show the correlation coefficient between the naming patterns.

**Table 1** presents our model’s simulation results averaged across 20 individual simulations. The correlations between language groups are presented in **Figure 4**: **Figure 4A** shows the empirical data reported by Ameel et al. (2005) and **Figure 4B** presented the averaged modeling results across 20 individual simulations. Similar to the pattern reported in Ameel et al. (2005), we found significantly higher correlations between the two languages in the bilingual model than between the two languages in the monolingual model (*Z* = 48.33, *p* < 0.0001). The higher correlations in the bilingual model, as compared with the monolingual model, also held for the two bilingual comparison models (*Z* = 38.90 and *Z* = 25.87, both *p*s < 0.001, for Comparison Models A and B, respectively). These simulation data indicate that all three models simulated empirical naming patterns and captured bilinguals’ convergence in lexical categorization.

**Table 1:** Results of model performance and the comparison between model performance and empirical data.

Model	Standard	Comparison Model A (no orthographic SOM)	Comparison Model B (no lateral connections)
Correlation between bilingual Dutch and bilingual French models	0.97	0.95	0.80
Correlation between empirical and simulation data in Dutch naming (bilingual model)	0.87	0.86	0.80
Correlation between empirical and simulation data in French naming (bilingual model)	0.83	0.78	0.76
Naming accuracy in the bilingual Dutch model	93%	89%	82%
Naming accuracy in the bilingual French model	92%	85%	86%

We then compared the correspondences between each model and the empirical data to identify the model that best matches the empirical data. **Table 1** presents the correlations between models and empirical data. These correlations reveal that the standard model produced results most similar to the empirical data. The z-transformed correlations were analyzed in a one-way ANOVA with 3 levels (type of models: Standard, Comparison A, and Comparison B). There was a significant main effect for type of models [*F*(2,57) = 155.88, *p* < 0.001, *F*(2,57) = 142.34, *p* < 0.001 for Dutch and French, respectively]. **Table 2** presents the results of Bonferroni-corrected *post hoc* comparison on the correlation of the name distribution and naming accuracy (see below for details on accuracy). The result showed that there were significant differences between each model pair on the correlation of the models’ name distributions to the empirical name distributions. These analyses confirm that both orthographic SOM and lateral connections were important in the model’s object naming. Also, the simulation data indicate that the role of lateral connection is more critical than the role of orthography.

**Table 2:** Pairwise comparison between models for correlation of the name distribution and naming accuracy.

			Standard Model vs. Model A	Standard Model vs. Model B	Model A vs. Model B
Correlation of name distribution	Dutch	*T*	5.19	14.02	13.61
		*P*	<0.001	<0.001	<0.001
	French	*T*	15.62	13.35	4.17
		*P*	<0.001	<0.001	<0.001
Naming accuracy	Dutch	*T*	13.45	18.29	11.95
		*P*	<0.001	<0.001	<0.001
	French	*T*	15.43	10.47	-0.75
		*P*	<0.001	<0.001	0.457

In the second measurement (see **Tables 1** and **2**), we examined the accuracy of dominant names produced by the model and compared it with the empirical data from Ameel et al. (2005). One-way ANOVAs with 3 levels (types of models: Standard, Comparison A, and Comparison B) showed that, for Dutch and French naming, there were significant main effects for type of models in both languages [*F*(2,57) = 233.29, *p* < 0.001, *F*(2,57) = 99.35, *p* < 0.001 for Dutch and French, respectively]. In **Table 2**, as with the results of correlation of the name distribution, analyses of the naming accuracy also indicated that the standard models produced results most similar to the empirical data, suggesting that both orthographic information and lateral connections were important in the model’s object naming. The role of lateral connection is more critical than the role of orthography in Dutch, but not in French.

### Semantic Convergence in the Model

Following Ameel et al. (2009), we first tested our model to examine if and how semantic convergence was manifested in our bilingual naming model.

#### Using Exemplar Typicality to Evaluate Category Convergence

**Table 3** presents the empirical results from Ameel et al. (2009) showing the correlations between objects’ mean typicality ratings in each category for monolinguals and bilinguals, along with the analogous results from our monolingual vs. bilingual models. The z-transformed correlations, as used earlier, were compared between monolingual and bilingual situations for each of the translation pairs. The analyses indicated that correlations in the bilingual model were significantly higher than in the monolingual model [*t*(38) = 73.74, *p* < 0.001 for *fles*–*bouteille*; *t*(38) = 17.07, *p* < 0.001 for *pot*–*pot*]. These findings are consistent with Ameel et al.’s (2009) empirical results and suggest that the corresponding categories for bilinguals are more similar across languages than the corresponding categories between monolinguals of each language.

**Table 3:** Correlations between mean typicality ratings for each pair of category names in the empirical results and simulation results (empirical data were adapted from Ameel et al., 2009).

	Pairs of categories		
	(Dutch–French)	Monolingual	Bilingual
Empirical results	*fles–bouteille*	0.91	0.98
	*pot–pot*	0.94	0.98
Simulation results	*fles–bouteille*	0.78	0.89
	*pot–pot*	0.82	0.96

#### Using Geometrical Position of Categories to Examine Category Convergence

Following the rationale discussed in Section “Category Centers: Geometrical Representation”, we used geometrical coordinates of objects to estimate the distance between categories and examined how convergence was manifested in the centers of lexical categories. Recall that boundary-dependent measures used the mean coordinates between the objects, and boundary-independent measures used the median coordinates. Thus, the boundary-dependent measures are supposed to be more influenced by objects at the boundaries of the category (see Category Centers: Geometrical Representation). A two-sample *t*-test was conducted between each of the selected category pairs across 20 simulations. The distance between category centers in the bilingual model was significantly less than the distance in the monolingual model [*t*(38) = 5.57, *p* < 0.001 for *fles*–*bouteille* pair; *t*(38) = 14.16, *p* < 0.001 for *pot*–*pot* pair] for both the boundary-dependent measure and [*t*(38) = 3.44, *p* < 0.001 for *fles*–*bouteille* pair; *t*(38) = 5.39, *p* < 0.001 for *pot*–*pot* pair] for the boundary-independent measure.

We also calculated the indirect-direct distance ratios to examine if bilingual prototypes were situated in between monolingual prototypes. A distance ratio equal to 1 indicates that the indirect distance equals the direct distance. Our analysis showed that all distance ratios are significantly greater than 1, suggesting that the bilingual category centers are not located in between the monolinguals category centers in our simulations.

These simulation results of category distances and distance ratios are partly consistent with Ameel et al. (2009). We demonstrated that the distances between corresponding category centers are less in the bilingual than in the monolingual model. Furthermore, because the measurement of boundary-independent centers also resulted in smaller distances in the bilingual simulations than in monolingual simulations, the convergence of the category centers in the bilingual situation cannot be due to the influence of boundary exemplars alone. We also showed that bilingual category centers are not directly in between the monolingual centers.

#### Boundary Complexity in Object Categories

As in Section “Category Boundaries: Complexity Level”, we compared complexity level between bilinguals and monolinguals to examine whether the convergence is manifested in boundary complexity. Recall that in Section “Category Boundaries: Complexity Level” we used the stepwise DFA to determinate the number of dimensions needed for each category pairs in each language groups. The number of dimensions required to separate two categories was used as index of complexity level of category relationship. The Wilks Lambda tests were significant in all final discriminant functions indicating that the selected variables in the final functions significantly differentiated between the two categories. The averaged canonical correlations across six category pairs (three in Dutch and three in French) in 20 simulations in the bilingual model and the monolingual model was 0.96 and 0.97, respectively. There is no difference between groups [*t*(119) = 1.087, *p* > 0.05]. We conducted a two-sample paired-*t* test for the average number of dimensions for the bilingual and monolingual simulations, to test whether or not the bilingual categories are linearly separable with the same number of dimensions as the monolingual categories. The statistical analysis showed that the number of dimensions in the final stepwise DFA was lower in the bilingual model than in the monolingual model, although this was only marginally significant [5.97 < 7.00, *t*(5) = 2.31, *p* = 0.069]. The results showed that the number of dimensions needed to distinguish categories is fewer for bilinguals than for monolinguals and suggested that bilinguals have less complex category relationships than monolinguals.

#### Examining Category Complexity through the Number of Category Outliers

We further examined whether bilinguals dropped the idiosyncratic items to form the less complex category relationships. **Table 4** presents the average proportions of outliers in each selected category name and language group. For example, for items in French *pot* category, 6% of them have closer distance to other categories in bilinguals and indicated that these 6% items were not categorized as French *pot* based on similarity. We compared number of outliers (divided by the total number of objects for the category) between bilinguals and monolinguals to reveal the composition of language groups. The bilingual model produced fewer outliers in 4 out of 6 categories. However, there was no statistically significant difference between the monolingual and the bilingual models in terms of the proportion of number of outliers. As such, we did not fully replicate the findings Ameel et al.’s (2009) Study 4 and therefore cannot argue that bilinguals dropped the idiosyncratic items to construct the less complex category relationships.

**Table 4:** Mean (and standard deviation) proportion of outliers of monolinguals and bilinguals for each selected category name.

	Category name	Bilinguals	Monolinguals
Dutch	*Fles*	0.24 (0.10)	0.20 (0.07)
	*Bus*	0.11 (0.11)	0.12 (0.11)
	*Pot*	0.12 (0.07)	0.12 (0.03)
French	*bouteille*	0.21 (0.16)	0.22 (0.15)
	*falcon*	0.23 (0.13)	0.37 (0.12)
	*pot*	0.06 (0.07)	0.20 (0.06)

## General Discussion

This study was aimed at investigating the mechanisms underlying the semantic convergence in the bilingual lexicon. By using a self-organizing connectionist network to simulate bilinguals’ object naming, we demonstrated that both lateral connections between languages and the orthographic information are important representations for semantic convergence. We also identified that the strength of name agreement of target and non-target languages enables the formation of complex relationships in the process of bilinguals’ object naming. Our study showed the advantages of computational modeling in examining the underlying mechanisms that are difficult to manipulate in empirical studies and provided a starting point for future work on bilinguals’ semantic convergence.

### Role of Lateral Connection and Orthographic Information

To identify the role of orthographic representations and lateral connections in bilingual lexical categorization, in this study we compared the standard models with comparison models (Model A and Model B) in which the critical mechanism was absent. Our simulations indicated that, when the model did not contain lateral connections or orthographic representations, its performance was significantly worse than the performance of a model in which lateral connections and orthographic representations were included. In addition, by comparing Models A and B, we found that lateral connections are more important than orthographic representations in simulating bilingual lexical categorization. Our model thus provides a starting point for studying a wide range of other learner and input variables that may influence behavioral outcomes for simultaneous and sequential bilinguals (e.g., age of onset, proficiency, and frequency of input).

Our model was designed to simulate the dynamic interactions between two languages and identified that lateral connections may play a critical role in bilingual lexical categorization. Our results demonstrate how, for simultaneous bilinguals, the processing of one language can be influenced by the other language (i.e., bi-directional influences between languages). Independent of any potential differences in the weights of object features-to-name correspondences across languages, lateral connections between languages have the potential to alter bilinguals’ naming patterns. In our simulations, these lateral connections resulted in convergent naming patterns between two languages. It is possible that the establishment of lateral connections for sequential bilinguals would be different from that for simultaneous bilinguals as we simulated in this study. For simultaneous bilinguals, the training of the lateral connections between the two languages started from an earlier time, whereas for sequential bilinguals, the training can only start after the learning of the L2. Elman (1993) showed that as learning progresses, weight changes in classic connectionist models tend to become less flexible and radical (the so-called ‘entrenchment’ effect; see Hernandez et al., 2005 and MacWhinney, 2016 for discussion). This property might also apply to our model if we were to simulate sequential bilinguals, for whom the semantic-phonological associations in the target languages may depend much more on the proficiency, exposure, effort, and the abilities/aptitudes of the learner (see also Malt et al., 2015).

Previous empirical work in lexical categorization has found significant cross-language transfer or convergence in bilinguals of languages that do not share orthographies (Russian and English: Pavlenko and Malt, 2011; Chinese and English: Zinszer et al., 2014; Malt et al., 2015). In these cases, convergence is not likely explained by orthographic similarities, and further work is needed to clarify this role of orthographic representations. Still, our model is the first to demonstrate the importance of orthographic similarity on naming common household objects, at least for languages that share significant similarity in orthography (such as Dutch and French). For Dutch–French simultaneous bilinguals, the naming model without orthographic representations also showed bilingual convergence in naming patterns, but it performed worse than the model with orthographic representations. Thus, orthography may be an important but not necessary cause for bilingual convergence. Further simulations will help to test the role of orthography in language pairs that have different degrees of orthographic similarity, and the present model’s method of encoding orthographic representations can be extended to such situations.

Another important finding from our simulations is that a word’s phonological activation level can be enhanced by orthographically similar words in the other language. This finding may shed some light on the cognate facilitation effect. Cognates are words that have identical or very similar orthographic/phonological features and identical or very similar meanings in two languages (Costa et al., 2000; Richards and Schmidt, 2002). In picture naming studies, several studies show that cognates are processed faster and more accurately than non-cognates (Costa et al., 2000; Gollan et al., 2005; Rosselli et al., 2012; Starreveld et al., 2013). Our simulations suggest that cognates received enhanced activation from the non-target language, which may drive faster processing in cognates than in non-cognate. Also, when correct picture names are determined by the translational equivalents (i.e., cognate names), the naming accuracy will be higher for cognates than non-cognates. However, as we mentioned in the Introduction, lexical categorization does not entail a one-to-one mapping across languages. A picture of an object may not evoke the translational equivalent names in the two languages. Further studies that directly compare picture naming patterns with versus without cognate names in the bilingual’s two language will be needed to gain a deeper understanding of the cognate effects.

### Bilingual Representation Convergence

Previous studies (Malt et al., 1999; Ameel et al., 2005) have demonstrated that speakers of different languages perceive objects similarly outside the context of naming. Those studies used object sorting data to calculate all objects’ pairwise similarity and found that the correlations of these pairwise similarities are very high between speakers of different languages with minimal group differences between monolinguals. Based on these studies, we constructed our model to use the same semantic feature vectors for Dutch monolinguals, French monolinguals, and Dutch–French bilinguals as input to the semantic SOM. Using these semantic feature vectors, our simulation replicated the basic findings reported in Ameel et al. (2005, 2009) and suggested that the underlying assumption that different language speakers (both monolinguals and bilinguals) use the same featural representations of objects is reasonable.

However, our simulation results differed from Ameel et al. (2009) in a few respects. First, the bilinguals’ category centers were not located in between monolinguals’ category centers (see Using Geometrical Position of Categories to Examine Category Convergence). This difference might be due to the way in which the two dimension-reduction methods of multidimensional scaling or MDS (used in Ameel et al., 2009) and SOM (used in our study) differ in adjusting the 2D spatial positions of vector representations: SOM capitalizes on the local similarities whereas MDS capitalizes on global dissimilarities (Kirt and Vainik, 2007), and SOM focuses on preserving the topology while MDS on preserving the distance (Moya-Anegón et al., 2006). Our simulation results based on category distances (local distance) were consistent with Ameel et al. (2009) because of the consistency of SOM and MDS analyses in this regard, but our simulation results based on the topographic relationships differed from the findings of Ameel et al. (2009). According to Ameel et al. (2009), bilingual category centers are situated in between monolingual category centers, perhaps because as bilingual proficiency increases the bilinguals’ categories move toward each other and away from monolinguals’ categories (i.e., ending in between the two monolingual languages’ categories). Our simulation suggest that bilingual convergence can result without the state of ‘in-betweeness’ for the bilingual prototypical representations as compared with monolingual prototypes.

The second difference between our simulation results and the empirical data of Ameel et al. (2009) was that in our simulations the monolingual and bilingual patterns could not be based on the number of feature dimensions needed (see Boundary Complexity in Object Categories), or the proportion of outliers (see Examining Category Complexity through the Number of Category Outliers). This discrepancy may be due to the small sample of objects and averaging bias. In Section “Boundary Complexity in Object Categories”, we compared the number of dimensions needed to discriminate between categories for monolinguals with bilinguals. Although the general trend was consistent with the empirical data (Ameel et al., 2009), there were only 6 pairs from the bottle set in the current simulation, as compared with 14 pairs in Ameel et al. (2009) that included objects of both bottles and dishware. In Section “Examining Category Complexity through the Number of Category Outliers”, we compared the proportion of outliers in the monolingual and bilingual simulations and found no significant difference. Comparing to Ameel et al. (2009), such differences were apparent probably due to the individual subject-based analyses adopted (no averaging of subject data), which was highly sensitive to outlier performance. In our simulations we did not run such individual simulation-based analysis given the strength and probability of name agreement in the data of each model. Overall, our result partially replicated the findings from Ameel et al. (2009) and suggested that the category relationships, as measured by the number of features needed, is less complex for bilinguals than for monolinguals.

### Factors Influencing Bilingual Lexical Categorization

Given that the standard model simulated empirical bilingual naming patterns the best, we next explore this model to under stand the factors that influence bilingual lexical categorization. As mentioned earlier, the activation levels for each object on the phonological SOM are the sum of three sources: semantic, phonological, and orthographic SOMs. We can study the contribution of these three sources by expressing the relationship among them as *ACTp* = *NA(T)* + *NA(N)* ×*W_tn_* + *Orth*, where *ACTp* stands for activation level on phonological SOM, *NA(T)* for name agreement of target language, *NA(N)* for name agreement of non-target language, *W_tn_* for the connection strength between languages (target and non-target language), and *Orth* for the orthographic similarity. This means that the activation levels of all candidate names on the phonological SOM in our model are determined by name agreement of languages, connection strength, and orthographic similarity. The candidate names with the highest activation level will be the output names.

Our simulation showed that the strength of name agreement, connection strength between languages, and orthographic similarity are important factors to determinate lexical naming patterns for bilinguals. While including the orthographic effect in the standard model significantly improved the simulation performance, in this section, we focus on the effect between words within a language. The effects of orthographic similarity are zero for most words, so we will discuss the influence of name agreement and connection strength. If the object has high name agreement in the target language, the influence from the non-target language through lateral connections cannot easily change its category. As expressed in the formula, when a candidate name in the target language has high *NA(T)*, its *ACTp* will be high and making it the winner of the output. If a candidate name has low *NA(T)*, its *ACTp* might be low and other candidate name could have higher *ACTp*. This results in an output response different from the target (native) language. This is consistent with Zinszer et al. (2014), who found that the level of agreement can predict the native-likeness of responses. However, our simulations further suggested that the level of activation interacts with strength of name agreement in the non-target language, in that high name agreement in non-target language can help to stabilize the naming performance in the target language, but only if there is a strong connection between names of the objects in two languages. As also expressed in the formula, this effect will be that when *Wtn* is high, *NA(N)* can influence *ACTp* and when *Wtn* is low, *NA(N)* has little effect on *ACTp*.

To see how the above works in detail, let’s imagine two candidate words (A and B) from the target language that correspond to one word (C) from the non-target language. A has strongest name agreement in the target language. The activation of A for bilingual *is (ACTa) = (A + C × CA)* where *CA* denotes the connection between items A and C in the two languages, and the activation of B is *(ACTb) = (B + C × CB)* where *CB* denotes the connection between items B and C in the two languages. In one scenario, if the connection strength *CA* is stronger than *CB*, the output will still be A (no change). In the second scenario, when the connection strength *CA* is weaker than *CB* while the *(B + C × CB)* is larger than *ACTa*, the output will change to B. In this case, the strength of B, C and CB will determine the output. If B is strong (only slightly weaker than A), a little boost from *C × CB* will help *ACTb* exceed *ACTa*, causing the output to change from A to B. If B is not so strong, even added strength in *C × CB* would not help *ACTb* to exceed *ACTa*, in which case the output will still be A. Finally, when there are more non-consistent word-to-word mappings (e.g., third pair *D-C*, etc.) between the target and non-target languages, the output will be influenced by the complex activation and connection weight patterns among the items of A–D, etc across the two languages. For example, consider an object named *fles* in monolingual Dutch and *bouteille* in monolingual French frequently. If the association between *fles* and *bouteille* is strong, when bilinguals are asked to name this object in Dutch, both the original association and the association from French would enhance *fles* as the output. By contrast, if the association between *fles* and *bouteille* is weaker than the association between *flacon* (Dutch) and *bouteille*, bilinguals may name the object *flacon* instead of *fles*. This pattern cannot be easily identified by behavioral and general statistical analyses (e.g., Zinszer et al., 2014) and showcases the advantage of computational modeling as presented in this study.

### Future Directions

The lexical categorization simulations for simultaneous bilinguals in this study make it possible to extend the model to other types of bilinguals. Using computational modeling, Zhao and Li (2013) showed that cross-language semantic priming could be successfully simulated in a connectionist model that incorporates Age of Acquisition (AoA) differences. When our model is extended to include effects of AoA and its interaction L2 proficiency, we will be in a better position to understand lexical categorization and bilingual object naming in sequential bilinguals.

With the promising outcomes for simulating categorization in adult second language learning and bilingual representation (see articles in the volume of Li, 2013), we can also extend our simulations to lexical categorization in child second language learning. In the current study, we did not simulate the development of lexical categories in monolinguals. We trained the model on empirical naming data from adults and simultaneous bilinguals only. How object naming patterns develop over time is an interesting issue for future research. Ameel et al. (2008) investigated the development of lexical categorization from 5, 8, 10, 12, 14 years olds through to adults. Early learning is affected by additional important factors, such as the age at which a new word is first encountered and what critical features children focus on for a particular object when using a category name.

As discussed earlier, De Groot (2014) contrasted two possible causes of observed bilingual convergence in phonology, grammar, and word use: underlying representations in the two languages that are more similar to one another than those of corresponding monolinguals’, or connections between two sets of perfectly monolingual-like representations such that cross-activation of representations of the two languages can result in one language having an influence on the behavioral output in the other. Our model was designed to be relevant to the cross-activation account. However, our simulation did not test the alternative account and cannot speak to the validity of it, or what role it may play along with any influence of cross-activation. Our model demonstrated that the structure and process of the parallel activation account can account for much of simultaneous bilinguals’ lexical naming convergence. Our model demonstrated that for simultaneous bilinguals, lateral connections on word form level could be established between words elicited by the objects. However, lateral connections in sequential bilinguals might be different from the situation in simultaneous bilinguals, and further studies will be needed to examine the processing of lateral connections or parallel activation in sequential bilingual situations.

We should also note that the current model simulated only one domain, household containers, which is part of the broader domain of artifacts. Like many artifact sub-domains, containers have a rich array of physical and functional features and their human creators can create almost infinite variations on them, which may make their lexical categorization particularly prone to differences across languages (see Malt and Majid, 2013 for discussion). However, even domains such as those of human action can entail considerably different lexical categorization patterns across languages (e.g., Saji et al., 2011); exploring the impact of different domain characteristics and the specific semantic relations between the two languages within the target domain remain for future work. Also, although our stimulus set was relatively large, it still cannot encompass the vast number of objects and competing names for them that an individual may be familiar with, nor did we simulate factors such as which language environment an individual is currently immersed in or whether they frequently engage in code-switching. These factors should be considered in future work when constructing a model simulating the development of lexical categories in both monolingual and bilingual populations.

## Conclusion

In this study, we successfully built a computational model of bilingual lexical categorization based on a connectionist SOM architecture. Similar architectures have previously been tested in other domains of language acquisition and processing (Zhao and Li, 2010, 2013; Kiran et al., 2013; Li and Zhao, 2013, 2015), and our model furthers this progress by simulating bilingual semantic convergence in the naming of common household objects as reported in the empirical literature (Ameel et al., 2005, 2009). We investigated the semantic convergence manifested in the centers and boundaries of bilingual lexical categories, analogous to the empirical comparisons in Ameel et al. (2009). Our model not only replicated these empirical findings but also identified how these effects may be computationally realized through the complex relationships of phonology, orthography, and semantics to modulate the dynamic interactions between two languages in the bilingual representation. This study, along with other emerging models, demonstrates the importance and utility of computational modeling for the study of dynamic patterns of cross-language lexical interactions in the bilingual mind.

## Author Contributions

PL and BM developed the theoretical concept and general model. SF performed the simulation and analyzed the data. SF and PL drafted and edited the manuscript, and BM and BZ commented on and revised the manuscript. All authors discussed the implications and agreed with the final version of the manuscript.

## Conflict of Interest Statement

The authors declare that the research was conducted in the absence of any commercial or financial relationships that could be construed as a potential conflict of interest.

## Footnotes

1 http://translate.google.com or http://fanyi.baidu.com/

2 We use ‘semantic’ here broadly to refer to conceptual knowledge associated with word forms in this study.

3 We used the same feature vector representations for all language groups given the evidence from the following studies. Malt et al. (1999) compared the perceived similarity among American English, Argentinean Spanish, and Mandarin Chinese using a sorting task. The pairwise similarities between objects were calculated for each language group. The correlations of similarity matrices between language groups were very high (above 0.9), demonstrating that different language groups perceived the objects similarly. See also Ameel et al. (2005) and Malt et al. (2011) for evidence of high agreement across language groups in perceived similarity for different stimulus sets.

4 Note that in the training process we did not label the nodes/units with any language membership identity information.

## References

[B1] Abutalebi J. Green D. (2007). Bilingual language production: the neurocognition of language representation and control. *J. Neurolinguistics* 20 242–275. 10.1016/j.jneuroling.2006.10.003

[B2] Ameel E. Malt B. Storms G. (2008). Object naming and later lexical development: from baby bottle to beer bottle. *J. Mem. Lang.* 58 262–285. 10.1016/j.jml.2007.01.006

[B3] Ameel E. Malt B. C. Storms G. Van Assche F. (2009). Semantic convergence in the bilingual lexicon. *J. Mem. Lang.* 60 270–290. 10.1016/j.jml.2008.10.001

[B4] Ameel E. Storms G. (2006). From prototypes to caricatures: geometrical models for concept typicality. *J. Mem. Lang.* 55 402–421. 10.1016/j.jml.2006.05.005

[B5] Ameel E. Storms G. Malt B. C. Sloman S. A. (2005). How bilinguals solve the naming problem. *J. Mem. Lang.* 53 60–80. 10.1016/j.jml.2005.02.004

[B6] Bi Y. Xu Y. Caramazza A. (2009). Orthographic and phonological effects in the picture-word interference paradigm: evidence from a logographic language. *Appl. Psycholinguist.* 30 637–658. 10.1017/S0142716409990051

[B7] Bowers J. S. Mimouni Z. Arguin M. (2000). Orthography plays a critical role in cognate priming: evidence from French/English and Arabic/French cognates. *Mem. Cogn.* 28 1289–1296. 10.3758/BF03211829 11219956

[B8] Cheng X. Schafer G. Akyürek E. G. (2010). Name agreement in picture naming: an ERP study. *Int. J. Psychophysiol.* 76 130–141. 10.1016/j.ijpsycho.2010.03.003 20227446

[B9] Costa A. Caramazza A. Sebastian-Galles N. (2000). The cognate facilitation effect: implications for models of lexical access. *J. Exp. Psychol. Learn. Mem. Cogn.* 26 1283–1296. 10.1037/0278-7393.26.5.1283 11009258

[B10] Correard M.-H. Grundy V. (eds) (2001). *The Oxford-Hachette French Dictionary*, 3rd Edn. Oxford: Oxford University Press.

[B11] De Groot A. M. B. (2014). “About phonological, grammatical, and semantic accents in bilinguals’ language use and their cause,” in *Multilingual Cognition and Language Use: Processing and Typological Perspectives*, eds Filipovic L. Putz M. (Amsterdam: John Benjamins Publishing Company), 229–262.

[B12] Dijkstra T. Grainger J. van Heuven W. J. B. (1999). Recognition of cognates and interlingual homographs: the neglected role of phonology. *J. Mem. Lang.* 41 496–518. 10.1006/jmla.1999.2654

[B13] Elman J. L. (1993). Learning and development in neural networks: the importance of starting small. *Cognition* 48 71–99. 10.1016/0010-0277(93)90058-4 8403835

[B14] French R. M. Jacquet M. (2004). Understanding bilingual memory. *Trends Cogn. Sci.* 8 87–93. 10.1016/j.tics.2003.12.011 15588813

[B15] Gollan T. H. Montoya R. I. Fennema-Notestine C. Morris S. K. (2005). Bilingualism affects picture naming but not picture classification. *Mem. Cogn.* 33 1220–1234. 10.3758/BF03193224 16532855

[B16] Hebb D. (1949). *The Organization of Behavior: A Neuropsychological Theory.* New York, NY: Wiley.

[B17] Hernandez A. E. Li P. MacWhinney B. (2005). The emergence of competing modules in bilingualism. *Trends Cogn. Sci.* 9 220–225. 10.1016/j.tics.2005.03.003 15866148 PMC4107303

[B18] Kan I. P. Thompson-Schill S. L. (2004). Effect of name agreement on prefrontal activity during overt and covert picture naming. *Cogn. Affect. Behav. Neurosci.* 4 43–57. 10.3758/CABN.4.1.43 15259888

[B19] Kiran S. Grasemann U. Sandberg C. Miikkulainen R. (2013). A computational account of bilingual aphasia rehabilitation. *Biling. Lang. Cogn.* 16 325–342. 10.1017/S1366728912000533 PMC3940390 24600315

[B20] Kirt T. Vainik E. (2007). “Comparison of the methods of self-organizing maps and multidimensional scaling in analysis of Estonian emotion concepts,” in *Proceedings of the 16th Nordic Conference on Computational Linguistics*, eds Nivre J. Kaalep H.-J. Muiscnek K. Koit M. (Tartu: University of Tartu), 113–120.

[B21] Kohonen T. (1982). Self-organized formation of topologically correct feature maps. *Biol. Cybern.* 43 59–69. 10.1007/BF00337288

[B22] Kohonen T. (2001). *The Self-Organizing Maps*, 3rd Edn. Berlin: Springer.

[B23] Kremin H. Hamerel M. Dordain M. De Wilde M. Perrier D. (2000). Age of acquisition and name agreement as predictors of mean response latencies in picture naming of French adults. *Brain Cogn.* 43 286–291. 10857710

[B24] Li P. (2013). Computational modeling of bilingualism: how can models tell us more about the bilingual mind? *Biling. Lang. Cogn.* 16 241–245. 10.1017/S1366728913000059

[B25] Li P. Farkas I. (2002). “A self-organizing connectionist model of bilingual processing,” in *Bilingual Sentence Processing*, eds Heredia R. Altarriba J. (North-Holland: Elsevier Science Publisher), 59–85.

[B26] Li P. Legault J. Litcofsky K. A. (2014). Neuroplasticity as a function of second language learning: anatomical changes in the human brain. *Cortex* 58 301–324. 10.1016/j.cortex.2014.05.001 24996640

[B27] Li P. MacWhinney B. (2002). PatPho: a phonological pattern generator for neural networks. *Behav. Res. Methods Instrum. Comput.* 34 408–415. 10.3758/BF03195469 12395557

[B28] Li P. Zhao X. (2013). Self-organizing map models of language acquisition. *Front. Psychol.* 4:828 10.3389/fpsyg.2013.00828 PMC3833256 24312061

[B29] Li P. Zhao X. (2015). “Computational modeling of bilingual language acquisition and processing: conceptual and methodological considerations,” in *Cambridge Handbook of Bilingual Processing*, ed. Schwieter J. W. (Cambridge: Cambridge University Press).

[B30] Li P. Zhao X. MacWhinney B. (2007). Dynamic self-organization and early lexical development in children. *Cogn. Sci.* 31 581–612. 10.1080/15326900701399905 21635309 PMC9808815

[B31] Lupker S. J. (1982). The role of phonetic and orthographic similarity in picture-word interference. *Can. J. Psychol.* 36 349–367. 10.1037/h0080652 7172126

[B32] MacWhinney B. (2016). “Entrenchment in second language learning,” in *Entrenchment, Memory and Automaticity: The Psychology of Linguistic Knowledge and Language Learning*, ed. Schmid H.-J. (Berlin: Mouton De Gruyter).

[B33] Malt B. C. Ameel E. Gennari S. (2011). “Do words reveal concepts,” in *Proceedings of the 33th Annual Conference of the Cognitive Science Society* (Austin, TX: Cognitive Science Society), 519–524.

[B34] Malt B. C. Li P. Pavlenko A. Zhu H. Ameel E. (2015). Bidirectional lexical interaction in late immersed Mandarin-English bilinguals. *J. Mem. Lang.* 82 86–104. 10.1016/j.jml.2015.03.001

[B35] Malt B. C. Majid A. (2013). How thought is mapped into words. *Wiley Interdiscip. Rev. Cogn. Sci.* 4 583–597. 26304265 10.1002/wcs.1251

[B36] Malt B. C. Sloman S. A. (2003). Linguistic diversity and object naming by non-native speakers of English. *Biling. Lang. Cogn.* 6 47–67. 10.1017/S1366728903001020

[B37] Malt B. C. Sloman S. A. Gennari S. Shi M. Wang Y. (1999). Knowing versus naming: similarity and the linguistic categorization of artifacts. *J. Mem. Lang.* 40 230–262. 10.1006/jmla.1998.2593

[B38] McCloskey M. E. Glucksberg S. (1978). Natural categories: well defined or fuzzy sets? *Mem. Cogn.* 6 462–472. 10.3758/BF03197480

[B39] McRae K. de Sa V. R. Seidenberg M. S. (1997). On the nature and scope of featural representations of word meaning. *J. Exp. Psychol. Gen.* 126 99–130. 10.1037/0096-3445.126.2.99 9163932

[B40] Miikkulainen R. (1997). Dyslexic and category-specific aphasic impairments in a self-organizing feature map model of the lexicon. *Brain Lang.* 59 334–366. 10.1006/brln.1997.1820 9299068

[B41] Miikkulainen R. Kiran S. (2009). Modeling the bilingual lexicon of an individual subject. *Cogn. Sci.* 5629 191–199.

[B42] Miller K. D. MacKay D. J. C. (1994). The role of constraints in hebbian learning. *Neural Comput.* 6 100–126. 10.1162/neco.1994.6.1.100

[B43] Moya-Anegón F. Herrero-Solana V. Jiménez-Contreras E. (2006). A connectionist and multivariate approach to science maps: the SOM, clustering and MDS applied to library and information science research. *J. Inform. Sci.* 32 63–77. 10.1177/0165551506059226

[B44] Osselton N. E. Hempelman R. (2003). *The New Routledge Dutch Dictionary*, 2nd Edn. London: Routledge.

[B45] Pavlenko A. Malt B. C. (2011). Kitchen russian: cross-linguistic differences and first-language object naming by Russian–English bilinguals. *Biling. Lang. Cogn.* 14 19–45. 10.1017/S136672891000026X

[B46] Rastle K. McCormick S. F. Bayliss L. Davis C. J. (2011). Orthography influences the perception and production of speech. *J. Exp. Psychol. Learn. Mem. Cogn.* 37 1588–1594. 10.1037/a0024833 21823810

[B47] Richards J. C. Schmidt R. (2002). *). Longman Dictionary of Applied Linguistics and Language Teaching*, 3rd Edn. Harlow: Longman.

[B48] Ritter H. Kohonen T. (1989). Self-organizing semantic maps. *Biol. Cybern.* 61 241–254. 10.1007/BF00203171

[B49] Rosselli M. Ardila A. Jurado M. B. Salvatierra J. L. (2012). Cognate facilitation effect in balanced and non-balanced Spanish-English bilinguals using the Boston Naming Test. *Int. J. Biling.* 18 649–662. 10.1177/1367006912466313

[B50] Saji N. Imai M. Saalback H. Zhang Y. Shu H. Okada H. (2011). Word learning does not end at fast mapping: evolution of verb meanings through reorganization of an entire semantic domain. *Cognition* 118 45–61. 10.1016/j.cognition.2010.09.007 21074145

[B51] Shook A. Marian V. (2013). The bilingual language interaction network for comprehension of speech. *Biling. Lang. Cogn.* 16 304–324. 10.1017/S1366728912000466 PMC3866103 24363602

[B52] Sirosh J. Miikkulainen R. (1994). “Self-organizing feature maps with lateral connections: modeling ocular dominance,” in *Proceedings of the 1993 Connectionist Models Summer School (CMSS-93, Boulder, CO)*, eds Mozer M. C. Smolensky P. Touretzky D. S. Elman J. L. Weigend A. S. (Hillsdale, NJ: Erlbaum), 31–38.

[B53] Starreveld P. A. De Groot A. M. B. Rossmark B. M. M. Van Hell J. G. (2013). Parallel language activation during word processing in bilinguals: evidence from word production in sentence context. *Biling. Lang. Cogn.* 17 1–19.

[B54] Wierzbicka A. (1992). *Semantics, Culture, and Cognition.* Oxford: Oxford University Press.

[B55] Zhang Q. Chen H.-C. Weekes B. S. Yang Y. (2009). Independent effects of orthographic and phonological facilitation on spoken word production in Mandarin. *Lang. Speech* 52 113–126. 10.1177/0023830908099885 19334418

[B56] Zhang Q. Weekes B. S. (2009). Orthographic facilitation effects on spoken word production: evidence from Chinese. *Lang. Cogn. Neurosci.* 24 1082–1096. 10.1080/01690960802042133

[B57] Zhao X. Li P. (2009). Acquisition of aspect in self-organizing connectionist models. *Linguistics* 47 1075–1112. 10.1515/LING.2009.038

[B58] Zhao X. Li P. (2010). Bilingual lexical interactions in an unsupervised neural network model. *Int. J. Biling. Educ. Biling.* 13 505–524. 10.1080/13670050.2010.488284

[B59] Zhao X. Li P. (2013). Simulating cross-language priming with a dynamic computational model of the lexicon. *Biling. Lang. Cogn.* 16 288–303. 10.1017/S1366728912000624

[B60] Zinszer B. D. Malt B. C. Ameel E. Li P. (2014). Native-likeness in second language lexical categorization reflects individual language history and linguistic community norms. *Front. Psychol.* 5:1203 10.3389/fpsyg.2014.01203 PMC4209811 25386149

